# Experimental, Genomic, and Structural Evidence Supporting Putative PET‐Hydrolases in Thermophilic Bacteria Isolated From Hot Springs in Cajamarca, Peru

**DOI:** 10.1002/mbo3.70376

**Published:** 2026-08-13

**Authors:** Marco A. Rivera‐Jacinto, Claudia Rodríguez‐Ulloa, Sara R. Briones‐Ramírez, Betsy Quispe‐Cruz, Sandra Carrasco‐Tarrillo, Diego A. Leonardo, Juan P. Cardenas

**Affiliations:** ^1^ Laboratorio de Microbiología, Departamento de Ciencias Biológicas Universidad Nacional de Cajamarca Cajamarca Perú; ^2^ Instituto de Física de São Carlos Universidade de São Paulo São Carlos São Paulo Brazil; ^3^ Departamento de Ciencias Biológicas, Facultad Ciencias de la Vida Universidad Andres Bello Santiago Chile

**Keywords:** comparative genomics, molecular docking, PET biodegradation, structure‐based enzyme prediction, thermophiles

## Abstract

Polyethylene terephthalate (PET) waste represents a major environmental challenge due to limited recycling solutions. Thermophilic bacteria from geothermal environments harbor diverse enzymatic machinery adapted to extreme conditions, offering promising biocatalysts for plastic degradation; however, biological resources from Peru and other South American countries remain scarce. We characterized four bacterial strains isolated from two geothermal sites in Cajamarca, Peru, screened for PET hydrolysis at 50°C. Whole‐genome sequencing using hybrid assembly achieved near‐complete circular genomes. GTDB‐Tk classification identified three species: *Neobacillus thermocopriae* (strain 19A), *Bacillus licheniformis* (strains 16P and BI2), and *Brevibacillus agri* (strain BI8). Quantitative assays revealed that strain 16P achieved the highest mass loss (0.598%), followed by strain BI8 (0.449%). ATR‐FTIR analysis of the incubated sheets showed a significant reduction of the ester carbonyl index in strains 16P, 19A, and BI8 relative to both non‐incubated PET and an abiotic control, whereas strain BI2 did not differ from the controls, indicating preferential modification of ester bonds at the sheet surface. Genome mining and structure‐based homology searches identified multiple candidate enzymes similar to validated PETases and carboxylesterases, including PETase46‐like homologs in strains BI8 and 16P and a terephthalate‐active carboxylesterase homolog in strain 16P. Molecular docking supported the conservation of catalytic geometry and substrate‐binding sites in these candidates. This work represents one of the first systematic genomic and structural characterizations of putative PET‐hydrolases in Peruvian geothermal bacteria, expanding knowledge of extremophile diversity and advancing thermostable enzymes for sustainable plastic waste management.

## Graphical abstract

Integrated microbiological, genomic, and structural analyses of bacteria isolated from a Peruvian geothermal system reveal enzymatic potential for the transformation of polyesters such as PET. This study links experimental evidence with genomic determinants and in silico analyses that support the thermophilic bacteria biodegradation capacity.

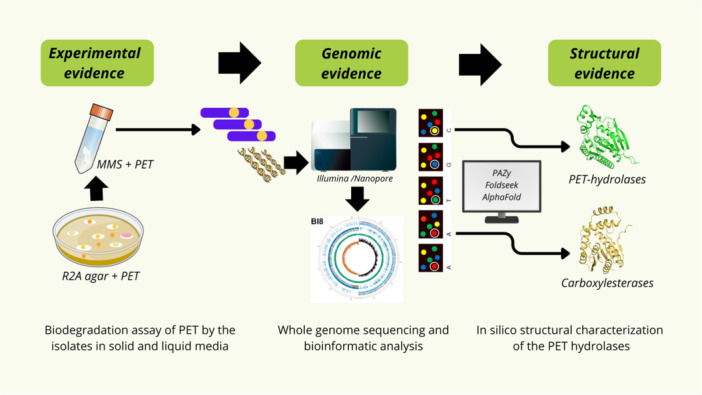

## 1 Introduction

Increasing plastic pollution is among the foremost environmental challenges of the 21st century, with profound implications for environmental and human health. In 2017, plastic production was estimated at over 8300 million metric tons worldwide; only a small fraction of those plastics has been recycled (Geyer et al. 2017). Among synthetic polymers, polyethylene terephthalate (PET) represents one of the most widely utilized plastics, representing approximately 9% of the global plastic waste volume (Alaraby et al. 2025). This semi‐crystalline polyester is extensively employed in the production of beverage bottles, food packaging, textiles, and various consumer products, resulting in the release of millions of metric tons of waste that can persist for several hundred years in natural environments (Alaraby et al. 2025). The environmental persistence of PET is primarily attributed to the stability of its ester bonds and the aromatic terephthalate monomers (Muringayil Joseph et al. 2024). Conventional disposal methods suffer from some important limitations such as loss of material quality, high energy requirements, and generation of toxic by‐products (Yao et al. 2022). Therefore, PET waste continues to accumulate in high volumes, releasing microplastics contaminating aquatic ecosystems and disrupting wildlife. Additionally, PET disposal by combustion represents a strong contributor to air pollution, with negative repercussions on biological and environmental health (Kováts et al. 2023).

In response to PET accumulation and pollution, the use of microorganisms and enzymes for PET degradation has emerged as a promising sustainable alternative. The discovery of PET‐hydrolyzing enzymes (PETases) represented a milestone in plastic bioremediation paradigms, offering a sustainable approach (Cai et al. 2023). Currently, most characterized PET‐hydrolases belong to the α/β‐hydrolase superfamily (particularly cutinases and carboxylesterases), catalyzing the hydrolytic cleavage of PET polymers into monomeric intermediates such as mono‐(2‐hydroxyethyl) terephthalate (MHET), bis(2‐hydroxyethyl) terephthalate (BHET), and ultimately terephthalic acid (TPA) and ethylene glycol, which can be reassimilated by microorganisms via different pathways (Taniguchi et al. 2019). Subsequent studies have revealed that validated PET hydrolases are found in thermophilic and mesophilic microorganisms from a variety of taxonomic phyla (mostly from Actinomycetes—currently Actinomycetota—and Proteobacteria—currently Pseudomonadota—) (Ahituv et al. 2025). PETases have been targeted for the generation of integral databases such as PAZy (Buchholz et al. 2022) or PlasticDB (Gambarini et al. 2022), or the use of protein discovery pipelines such as VenusMine (Wu et al. 2025). Various types of hydrolase enzymes have been associated with the hydrolytic capacity of different bacteria and fungi on plastics (Pérez‐García et al. 2025; Temporiti et al. 2022; Roberts et al. 2020): among the described enzymes are several types of proteases, laccases, ureases, and esterases (Pérez‐García et al. 2025).

Despite the fact that the first PETase activity was found in *Ideonella sakaiensis*, a mesophilic bacterium (Yoshida et al. 2016), PETases have also been associated with thermophilic bacteria (Hu et al. 2025). This feature is interesting, considering that thermophilic organisms living in environments such as hot springs, have been considered interesting “living enzyme factories” (Burkhardt et al. 2023). Hot springs represent unique extreme environments that harbor taxonomically and metabolically diverse thermophilic microbial communities. These geothermal ecosystems, characterized by elevated temperatures (45°C–90°C), variable pH, and high mineral content, provide selective pressures that drive the evolution of specialized enzymatic machinery capable of functioning under harsh physicochemical conditions (Urbieta et al. 2023). The exploration of hot spring environments for novel biocatalysts has proven remarkably successful worldwide, with thermostable enzymes including cellulases, xylanases, amylases, proteases, lipases, and hydrolases being discovered from diverse geothermal sites (Burkhardt et al. 2023; Ulucay et al. 2022). These extremozymes demonstrate exceptional stability and activity at high temperatures, making them invaluable for biotechnological applications ranging from biofuel production to industrial waste treatment (Littlechild 2015).

In South America, the functional diversity of different hot spring systems has been scarcely analyzed. Geothermal systems distributed along the Andean mountain cordillera have emerged as significant reservoirs of microbial biodiversity and biotechnological potential. Bacterial isolation/characterization from different South American sites have been previously reported for geothermal systems in Copahue, Argentina (Willis et al. 2019; Urbieta et al. 2015; Giaveno et al. 2013), Atacama, Chile (Valenzuela et al. 2024; Muñoz‐Torres et al. 2023), Colombian Andes (Rubiano‐Labrador et al. 2019), or Papallacta, Ecuador (Arias et al. 2016). In Peru, several efforts to characterize and utilize thermophilic microorganisms with biotechnological purposes were published. Isolates obtained from different zones in the Cajamarca region (Valdez‐Nuñez and Rivera‐Jacinto 2024) were evaluated in their abilities of biosurfactant production, siderophore synthesis, and PET hydrolysis. Other studies using isolates (Tamariz‐Angeles 2014) and microbial communities of thermophilic bacteria from hot springs in Huancarhuaz (Ancash region) were found with abilities for lignocellulose bioconversion (Castañeda‐Barreto et al. 2024). A set of some Gram‐positive bacteria was isolated and characterized from different hot springs in Tacna (Ticona et al. 2025) and Carhuaz (Tamariz‐Angeles et al. 2020). The characterization of biotechnological properties of thermophilic microorganisms, including their potential for PET degradation, can be addressed by both experimental and genomic approaches, including the essential role of whole‐genome sequencing (WGS) (Ticona et al. 2025; Castañeda‐Barreto et al. 2024).

The following study aims to conduct WGS analysis of bacterial strains isolated from hot springs in Cajamarca, Peru. We will emphasize the identification and characterization of genetic determinants associated with putative PET‐hydrolases production. By combining cultivation‐based isolation, experimental assessment of PET degradation by mass loss and attenuated total reflectance–Fourier transform infrared spectroscopy (ATR‐FTIR) spectroscopy, high‐throughput sequencing technologies and bioinformatic analyses, our work aims to present the genomic and taxonomic properties of those strains, to identify PET‐hydrolase candidate genes and related catabolic pathways through genome mining. Additionally, we will present a comparative genomic analysis with previously described members of the same species and we will make an *in silico* analysis of the structure and function of those enzymes. This work aims to contribute as a pioneering study to systematically explore the biocatalytic resources of Peruvian geothermal ecosystems for plastic management, contributing both to fundamental knowledge of extremophile diversity and to the development of bioremediation technologies addressing the global plastic pollution crisis.

## 2 Methodology

### 2.1 Screening for PET Hydrolase Activity and Characterization of Thermophilic Bacteria

All recovered isolates were inoculated on solid medium supplemented with PET using the double‐layer technique. The first layer consisted of 20 mL of R2A agar (Merck Millipore, Darmstadt, Germany) and the second layer of 20 mL of solid medium containing 0.0075% (w/v) PET powder (Charnock 2021). The cultures were incubated at 50°C for 72 h, and growth and halo formation were monitored every 24 h by measuring their diameter. The bacterial isolates were characterized on Columbia nutritive agar (Oxoid, Basingstoke, UK). The microscopic morphology of the cells was established using Gram staining. The characterization of the bacterial colonies included the morphological description of the colony diameter (in mm), shape, edge, elevation, texture, surface appearance, transparency, and pigment production.

### 2.2 Metabolic Characterization of Isolates

Metabolic activity was characterized using differential tests, including citrate utilization as the sole carbon source on Simmons citrate agar; sugar fermentation on TSI (Triple Sugar Iron) agar; lysine decarboxylation on LIA (Lysine Iron Agar); ornithine decarboxylation, indole production, and motility on MIO (Motility Indole Ornithine) agar; and indole and motility detection on SIM (Sulfide Indole Motility) agar. MR‐VP (Methyl Red–Voges‐Proskauer) broth was used to determine the metabolic pathway involved in glucose fermentation, and urea agar was used to evaluate the ability to utilize urea as the sole nitrogen source. All culture media were obtained from Merck Millipore. Additionally, catalase and cytochrome oxidase tests were performed using oxidase strips (Oxoid), and hemolysis was evaluated on 5% blood agar (Merck Millipore).

### 2.3 Tolerance to Different NaCl Concentrations and Temperatures

Isolates were inoculated in nutrient broth (Oxoid) supplemented with NaCl at 1.5%, 3%, and 7% to evaluate salt tolerance. The culture tubes were incubated at 50°C for 24–48 h. The strains were also inoculated in BHI broth (Oxoid) to assess growth at 18°, 45°, 50°, and 65°C for 24 h. All experiments were performed in triplicate, and growth was measured visually by comparing the culture tubes with an uninoculated tube maintained under the same incubation conditions.

### 2.4 Qualitative Characterization of Bacterial Enzymatic Activity

The production of β‐glucosidase (EC 3.2.1.‐), which cleaves the glycosidic bond between esculin and its sugar group (glucose), was evaluated using Esculin Agar medium (Sigma‐Aldrich, St. Louis, MO, USA). The activity endo‐exo‐glucanase (EC 3.2.1.4) was evaluated on carboxymethyl cellulose agar (CMC agar). The medium was prepared using a modified basal formulation containing 0.5% yeast extract (Oxoid), 0.1% KH_2_PO_4_ (Sigma‐Aldrich), and 1.5% agar (Difco, Detroit, MI, USA), supplemented with 1% CMC (Sigma‐Aldrich) as the sole carbon source. Other bacterial enzymatic activities were screened using Nutrient Agar (Merck Millipore) as the basal medium, supplemented with specific substrates for each assay. Starch (1% w/v) was added to evaluate alpha‐amylase (EC 3.2.1.1) production, while casein (1% w/v) was used for serine proteases (EC 3.4.21.‐). Lipolytic activity (EC 3.1.1.3) was determined by supplementing the basal medium with either tributyrin, Tween 80, or egg yolk (lecithinase). The production of gelatin metalloproteases (EC 3.4.24.‐), which are metal‐dependent proteases, was also evaluated using Nutrient Gelatin medium. Formation of clear halos around colonies was indicative of positivity.

### 2.5 PET Biodegradation Assay

Each bacterial isolate was inoculated into synthetic medium (MS) composed of (g·L^−1^): NH_4_NO_3_, 1.0; MgSO_4_·7H_2_O, 0.2; K_2_HPO_4_, 1.0; CaCl_2_·2H_2_O, 0.1; KCl, 0.15; and 1.0 mg·L^−1^ of each of the following trace elements: FeSO_4_·6H_2_O, ZnSO_4_·7H_2_O, and MnSO_4_ (Hadad et al. 2005). All chemical components of the medium were acquired from Sigma‐Aldrich. The medium was filter‐sterilized, adjusted to pH 7, and supplemented with PET sheets (0.77 ± 0.03% [w/v]). The inoculum represented 10% of the total medium volume, with a final cell concentration of 1.5 × 10^7^ CFU·mL^−1^, estimated by spectrophotometry in UNICO 2100 spectrophotometer (United Products & Instruments Inc., USA) and plate counting on Tryptone Soya Agar (Oxoid). Cultures were incubated at 50°C in a shaker at 190 rpm for 4 weeks. Experiments were carried out by triplicate, including a flask with uninoculated MS + PET medium (abiotic control). At the end of the incubation period, the PET sheets were recovered from the MS medium, rinsed with ultrapure water, and dried prior to determining their final mass. To minimize measurement error, each sheet was weighed in triplicate (technical replicates), and the three values were averaged to obtain a single representative datum for each biological replicate. The percentage of PET sheet mass loss was calculated as follows: % mass loss = [(initial mass − final mass)/initial mass] × 100.

Results were expressed as mean ± standard deviation of the three biological replicates per group. In addition, the net percentage of mass loss was calculated by subtracting the average loss observed in the abiotic control, propagating uncertainty through the square root of the sum of the variances of both groups (SD_net = √(SD^2^bacteria + SD^2^control)). Differences among the evaluated groups (bacterial strains and abiotic control) were analyzed using one‐way analysis of variance (ANOVA). Post‐hoc pairwise comparisons were performed using Tukey's test for multiple comparisons, considering differences statistically significant at *p* < 0.05. Statistical analyses were conducted using SPSS v.27.

### 2.6 FTIR‐ATR Analysis of PET Sheets

PET sheets (1 × 1 cm) were incubated for 1 month in mineral salts medium inoculated with each bacterial strain isolate. Non‐incubated PET (P) and PET incubated in MS medium without bacterial inoculum (PET abiotic control) were included as initial and abiotic controls, respectively. After incubation, the sheets were washed, dried, placed in clean tubes and sent for analysis. Three independent replicates were prepared per strain and both faces of each sheet were measured, yielding six spectra per strain and two spectra per control. Spectra were acquired on a Shimadzu IRTracer‐100 FTIR spectrophotometer (Shimadzu Corporation, Kyoto, Japan) equipped with an ATR accessory, using 20 scans, a resolution of 2 cm^−1^, Sqrt‐Triangle apodization and a spectral range of 4000–400 cm^−1^. Before each measurement the ATR crystal was cleaned and an instrumental blank was recorded without sample. Each sheet was placed directly on the ATR crystal and pressed with the clamp. Spectra were processed with the instrument software to reduce moisture and CO_2_ interferences, and transmittance was converted to absorbance as A = 2 ‐ log_10_(%T). Because the absolute band intensities in ATR depend on the degree of sheet‐crystal contact, which varies between independent measurements, all spectra were normalized to the aromatic ring band at 1409 cm^−1^. This in‐plane ring mode is insensitive to ester bond cleavage and to environmental aging, and has therefore been established as an internal reference for the normalization of PET spectra (Rostampour et al. 2024). From the normalized spectra, a carbonyl index (A_1713_/A_1409_) and an ester index (A_1242_/A_1409_) were calculated for each measurement, where the band at 1713 cm^−1^ corresponds to the ester C═O stretching and the band at 1242 cm^−1^ to the ester C–O stretching of PET. A decrease in these indices relative to the PET controls was taken as an indication of ester bond cleavage at the sheet surface. Differences between each strain and the pooled PET controls (P + PET abiotic control) were assessed by a two‐tailed Welch's *t*‐test for unequal variances, with *p* < 0.05 considered statistically significant. Values are reported as mean ± standard deviation.

### 2.7 Genomic DNA (gDNA) Isolation

For gDNA isolation, a pure single colony of each isolate was inoculated into 5 mL of Luria Broth (Oxoid) and incubated at 50°C for 16 h. gDNA was extracted using the ZymoBIOMICS DNA Miniprep Kit (Zymo Research Corporation, CA, USA) following the manufacturer's instructions. DNA quality and quantity were assessed by agarose gel electrophoresis and Nanodrop spectrophotometry (Thermo Fisher Scientific, MA, USA) to ensure integrity, concentration, and purity suitable for library construction and sequencing.

### 2.8 PCR Amplification of 16S rRNA Gene

The bacterial 16S rRNA gene was amplified using universal oligonucleotide primers 27F (5′‐AGA GTT GAT CCT GGC TCAG‐3′) and 1492R (5′‐TAC GGY TAC CTT GTT ACG ACTT‐3′) (Uluçay 2025). PCR mixture used for amplification contained: 1 μL DNA, 5 μL 10X buffer, 5 μL 2 mM dNTPs, 1.5 μL of each primer (10 μM), 25 mM MgSO_4_, 1 μL KOD Hot Start DNA Polymerase (1 U/μL), and PCR grade water to reach a final volume of 50 μL. PCR was conducted using a S1000 Thermal Cycler (Bio‐Rad Laboratories Inc., USA). PCR setup consisted of 35 cycles of denaturation at 95°C for 20 s, annealing at 54°C for 30 s, elongation at 70°C for 20 s and a final extension at 70°C for 5 min. PCR products were loaded in 1% (w/v) agarose gel to check the size of the amplicons through electrophoresis. Amplicons were visualized in Visi‐Blue Transilluminator (UVP, Analytik Jena, Jena, Germany) and stored until sequencing.

### 2.9 Genomic DNA Extraction, Library Construction and WGS

Genomic DNA was extracted from overnight cultures using the ZymoBIOMICS DNA Miniprep Kit following the manufacturer's protocol. DNA quality was verified by Nanodrop spectrophotometry (A260/A280 > 1.8) and Quantus fluorometry (concentration > 50 ng/μL). For Nanopore sequencing, library preparation was made with 200 ng of gDNA per sample. Fragment barcoding was performed using the Rapid Barcoding Kit 24 V14 (SQK‐RBK114.24) which contains barcodes RB01‐24 (FLO‐MIN114) (Oxford Nano­pore, Oxford, UK). For Illumina sequencing, the genomic libraries were prepared with the Nextera DNA XT Library Preparation Kit, which enables enzymatic fragmentation of DNA and simultaneous adapter ligation through transposition (Illumina, San Diego, CA, USA). Libraries were sequenced using the Illumina NovaSeq X platform, generating 150 base pair paired‐end reads (150 bp PE), performed to a depth of approximately 2 Gb per sample, ensuring sufficient coverage for downstream genomic analysis. Oxford Nanopore sequencing was carried out using R10.4.1 flow cells on a MinION device, achieving > 100× coverage.

### 2.10 Bioinformatic Analyses

Illumina sequences were filtered using Trimmomatic using as relevant parameters “PE AVGQUAL:25 LEADING:25 TRAILING:25 SLIDINGWINDOW:4:15 MINLEN:145 HEADCROP:19” (Bolger et al. 2014). In the case of Nanopore data, raw data were base‐called with Guppy v6.0 (https://nanoporetech.com/document/Guppy-protocol) in high‐accuracy mode. Nanopore reads were filtered using Nanofilt, with the relevant parameters “–headcrop 75 ‐q 12 ‐l 1000” (De Coster et al. 2018). Hybrid assembly of second‐ and third‐generation reads was done with Unicycler v0.5.0 (Wick et al. 2017), followed by polishing with Medaka v1.4.3 (https://github.com/nanoporetech/medaka). Polished assemblies were checked for contamination using CheckM2 (Chklovski et al. 2023). The taxonomic identity at the genomic level was confirmed using the program “classify_wf” of the GTDB‐Tk program, version 2.4.0 (Chaumeil et al. 2022), using the database release 220 as the reference. Genome annotation was performed using Prokka v1.14.6 with default parameters (Seemann 2014). Functional annotation was performed with different tools: eggNOG‐mapper was used with the parameters: “–tax_scope_mode narrowest –tax_scope prokaryota_broad –go_evidence experimental” (Cantalapiedra et al. 2021). Carbohydrate‐active enzymes (CAZymes) were predicted using the CAZy database as a reference, via run_DBCAN3, recovering only sequences with simultaneous hits in three different tool references (hmmsearch, diamond, dbCAN_sub) (Zheng et al. 2023). In order to search for antimicrobial resistance markers, the predicted ORFeome was analyzed by the *Resistance Gene Identifier* tool from the CARD database (*RGI‐main* 6.0.3, CARD 4.0.0), considering perfect and strict hits only (Alcock et al. 2023). Secondary metabolite biosynthetic clusters were searched in the assemblies using AntiSMASH v7.0 (Blin et al. 2023). Subcellular localization predictions using ORFeomes were performed by PSORTb 3.0 for Gram‐positive bacteria (Yu et al. 2010).

In order to make a phylogenetic tree for the bacterial strains, the 16S rRNA gene sequences were retrieved from the Prokka annotations and compared using BLAST against the NCBI *refseq_rna* database to obtain information from phylogenetically closest bacterial strains (Goldfarb et al. 2025). Combined sequences were aligned using MAFFT version 7.490 (parameters: “–maxiterate 1000 –localpair”) (Katoh 2002). The alignment was used by *iqtree* version 3.0.1 (parameters: “‐m TEST –alrt 1000 ‐B 1000”) to generate a maximum likelihood‐based tree (Wong et al. 2026) with a simultaneous aLRT (also known as the SH‐like test) and ultrafast bootstrap, each with 1000 replicates, as branch support tests. The “‐m TEST” parameter activated the standard model selection mode to search the substitution model with the best likelihood according to the Bayesian Information Criterion (BIC) (Sullivan 2005); the best model found was the TIM3 (variable base frequencies, variable transition rates considering AC = CG and AT = GT), considering the proportion of invariable sites and applying a discrete Gamma model with four categories (TIM3 + I + G4). The visualization for the phylogenetic tree was generated using the *ggtree* R package (Xu et al. 2022).

### 2.11 *In Silico* Structural Characterization of the PET Hydrolases

Protein sequences potentially involved in plastic degradation were initially selected from curated databases specialized in plastic‐degrading enzymes, including PAZy (Buchholz et al. 2022) and PlasticDB (Gambarini et al. 2022). Amino acid sequences corresponding to putative hydrolases and esterases were retrieved and used as input for subsequent structural and comparative analyses. An initial exploration was performed on the basis of sequence similarity by performing Diamond v. 2.1.8 (Buchfink et al. 2015) searches against the PlasticDB/PAZy reference (relevant parameters: “–ultra‐sensitive –*E*‐value 0.00001 –matrix BLOSUM45”). Hits obtained by this method were used to identify structural homologs and prioritize candidates with potential relevance to plastic degradation pathways. A structure‐based homology search was performed using *Foldseek* (https://search.foldseek.com) (van Kempen et al. 2024). This software enables fast and sensitive protein structure comparisons by encoding three‐dimensional protein structures into sequences based on a structural alphabet that captures tertiary residue–residue interactions, allowing efficient detection of remote structural homologs at a scale comparable to sequence‐based searches. This approach provides improved sensitivity for identifying evolutionary and functional relationships that may not be evident from sequence similarity alone (van Kempen et al. 2024). Structural matches identified by *Foldseek* were used as a filtering criterion to retain proteins exhibiting similarity to enzymes previously reported to participate in polyester or aromatic ester hydrolysis. Following candidate selection, *de novo* structural models of the filtered protein set were generated using the AlphaFold Server (https://alphafoldserver.com), which implements AlphaFold 3 (Abramson et al. 2024). Default parameters were used for all predictions, and only models with high overall confidence were considered for downstream analyses.

### 2.12 Molecular Docking Analysis

Molecular docking simulations were subsequently carried out to explore potential ligand‐binding sites and interactions with PET‐related substrates. Docking calculations were performed using AutoGridFR v1.0 (Zhang et al. 2019). The AutoSite module was employed to identify and cluster high‐affinity points within putative ligand‐binding pockets, and the docking grid box was positioned accordingly. Affinity maps were generated using default parameters, and docking simulations were conducted using a randomized docking protocol with eight independent runs (‐nbRuns 8), each allowing up to 200,000 scoring function evaluations (‐maxEvals 200,000). Residues involved in ligand interactions were identified through visual inspection and interaction analysis using Discovery Studio Visualizer v21.1.0 (BIOVIA, Dassault Systèmes). Structural figures were generated using PyMOL v3.1.3.1 (Schrödinger, LLC, New York, NY, USA). To assess sequence conservation and validate the structural relevance of residues implicated in ligand binding, multiple sequence alignments were performed using Jalview (Waterhouse et al. 2009), employing the Clustal algorithm with default parameters. Alignments included the candidate sequences and their closest structural homologs identified by *Foldseek*, enabling evaluation of residue conservation within predicted active or ligand‐binding sites.

## 3 Results

### 3.1 Macroscopic, Microscopic, and Phenotypic Evaluation of the Isolates

The diameter of the bacterial colonies ranged from 1 to 8 mm. Irregular, filamentous, and rhizoid colonies were observed. The colonies were primarily lobed, filamentous, and wavy. The colonies were raised, and only strain 16P presented umbilicated colonies. All colonies were opaque and cream‐colored. Furthermore, most isolates had a shiny surface, and only isolates BI2 and 16P had a dry texture (Table 1). The bacterial isolates were evaluated based on their biochemical and metabolic characteristics in the different differential media (Table 2); this data allowed the classification of each isolate at the genus level and revealed variations in their metabolic profiles. The tributyrin hydrolysis assay by each isolate was established as the primary detection of esterase/lipase activity, enzymes associated with the process of PET degradation mediated by microorganisms, which enabled the continuation of the biodegradation assay for each isolate. This assay showed that all four strains possessed this ability, with a visible activity in BI8 and 16P strains (Figures S1, SA2, SA4).

**Table 1:** Microscopic and macroscopic morphological characteristics of the isolates.

	Strain			
Characteristic	BI2	BI8	19 A	16 P
Gram stain	Positive	Positive	Positive	Positive
Cell morphology	Medium‐sized bacilli	Small bacilli	Short bacilli	Short bacilli
Endospore	Present	Present	Present	Present
Colony size (mm)	3.5 × 4	8 × 7	1	2
Colony shape	Rhizoid	Rhizoid	Irregular	Filamentous
Edge	Undulate	Lobate	Undulate	Filamentous
Elevation	Raised	Raised	Raised	Umbilicate
Texture	Dry	Smooth	Smooth	Dry
Surface	Rough	Shiny	Shiny	Rough
Transparency	Opaque	Opaque	Opaque	Opaque
Pigment	Cream	Cream	Cream	Cream

**Table 2:** Biochemical, metabolic characteristics, and enzymatic activity of the isolates.

		Strain			
Tests		BI2	BI8	19 A	16 P
Sugar fermentation	Glucose	+	−	−	+
	Lactose	+	−	—	+
	Sucrose	+	−	—	+
Lysine decarboxylation		−	−	—	—
Lysine deamination		—	—	—	—
Ornithine decarboxylation		—	NG	—	—
Citrate utilization		—	—	—	—
Motility		+	—	—	+
Indol production		—	—	—	‐+
Methyl Red		+	+	+	+
Voges‐Proskauer		—	—	—	—
Urea utilization		—	NG	—	—
Tellurite reduction		+	+	+	+
Cytochrome oxidase		—	+	—	—
Catalase production		+	—	+	+
Hemolysin		β	β	β	β
Hydrolysis	Gelatin	—	—	NG	—
	Casein	—	—	+	—
	Starch	—	—	—	—
	Tributyrin	+	+	—	+
	Lecithin	—	+	+	—
	CMC	+	—	—	+
	Esculin	+	—	—	+
	Tween 80	—	+	—	+
Growth in NaCl	1.5%	+	+	—	+
	3%	+	—	—	+
	7%	+	—	—	+
Growth at temperature	18°C	+	+	—	+
	45°C	+	+	+	+
	50°C	+	+	+	+
	65°C	—	—	—	—

*Note:* +: positive, –: negative, NG: no growth, β: beta‐hemolysis, CMC: carboxymethylcellulose.

### 3.2 Evaluation of PET Degradation by Growth, Mass Loss, and ATR‐FTIR Analysis

The four screened strains exhibited the ability to grow on solid medium containing 0.0075% PET and in liquid medium supplemented with PET as the only carbon source (Table S1). After 6 days of incubation on solid medium at 50°C, with evaluations performed every 24 h, positive growth was observed. On the first day, colonies reached a diameter of approximately 1 mm, indicating initial growth at the inoculation point. Progressive increases in colony size were recorded for isolates BI2 and 16P, whereas isolates BI8 and 19A maintained their diameters (Figure S1B). These results indicate that the isolates are able to grow with 0.0075% PET as the sole carbon source, although at a slower rate than in enriched media. In liquid medium, the population density of each isolate remained stable (~ 1.5 × 10^7^ CFU/mL). After 4 weeks of incubation at 50°C, PET sheet mass loss was determined (*n* = 3, independent replicates per strain). Sheets incubated in MS medium without bacterial inoculum were included as abiotic controls (*n* = 3). To account for variations in initial sheet weight, mass loss values were normalized and expressed as the percentage of weight loss relative to the initial mass. For each biological replicate, technical triplicates were averaged to minimize weighing instrument error. Mass measurements were performed on a balance with a sensitivity of 0.1 mg. Isolate 16P exhibited the highest mass loss (0.598 ± 0.051%), followed by BI8 (0.449 ± 0.07%), the values for 19A and BI2 were 0.209 ± 0.008% and 0.287 ± 0.032%, respectively (Table S1). Mass loss differed significantly among groups (one‐way ANOVA, *F* = 85.5, *p* < 0.001). All four strains showed significantly higher mass loss than the abiotic control (post‐hoc pairwise comparisons, *p* < 0.01), confirming that the observed mass reduction was biologically mediated rather than a result of abiotic degradation. Among the strains, 16P showed the highest mass loss, significantly greater than that of all other isolates (*p* < 0.05), while the difference between BI2 and 19A was not statistically significant (*p* = 0.23) (Figure S2).

**Figure 1:**
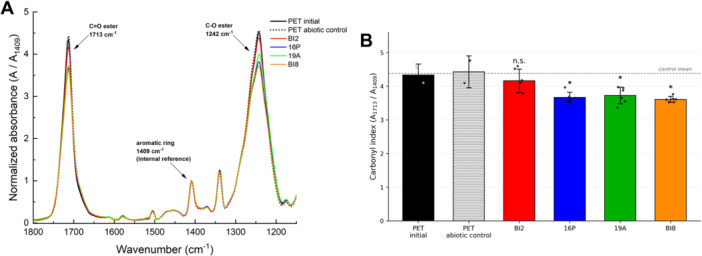
ATR‐FTIR analysis of PET sheets after 1 month of incubation with the four bacterial isolates. (A) Mean ATR‐FTIR spectra (1800–1150 cm^−1^) of PET sheets, normalized to the aromatic ring band at 1409 cm^−1^ used as internal reference. The main PET bands are indicated: ester C═O stretching (~1713 cm^−1^), ester C–O stretching (~1242 cm^−1^) and the aromatic ring mode used for normalization (~1409 cm^−1^). Non‐incubated PET (PET initial) and PET incubated without bacterial inoculum (PET abiotic control) are shown together with sheets incubated with strains BI2, 16P, 19A, and BI8. (B) Carbonyl index (A_1713_/A_1409_) for each group; bars represent the mean and error bars the standard deviation, with individual measurements overlaid (*n* = 6 spectra per strain, from three independent replicates measured on both sheet faces; *n* = 2 for each control). The dashed line indicates the mean carbonyl index of the pooled PET controls. Asterisks denote a significant reduction relative to the pooled PET controls (Welch's *t*‐test, *p* < 0.05); n.s., not significant.

Given the low magnitude of the mass loss values, ATR‐FTIR spectroscopy was used to determine whether chemical modification of the PET ester bonds had occurred at the sheet surface. All spectra displayed the characteristic PET bands, including the ester carbonyl stretching (~ 1713 cm^−1^), the ester C–O stretching (~ 1242 cm^−1^), and the aromatic ring modes (~ 1409 and ~ 1504 cm^−1^) (Figure 1A). Because absolute band intensities in ATR depend on the degree of sheet‐crystal contact, all spectra were normalized to the aromatic ring band at 1409 cm^−1^, an in‐plane ring mode insensitive to ester cleavage that serves as an internal reference (Rostampour et al. 2024). After normalization, the sheets incubated with strains 16P, 19A, and BI8 showed a significant reduction of the carbonyl index (A_1713_/A_1409_) relative to the pooled PET controls, corresponding to reductions of 16.3% (3.67 ± 0.15; *p* = 0.018), 14.9% (3.73 ± 0.25; *p* = 0.020) and 17.6% (3.61 ± 0.09; *p* = 0.017), respectively; control values were 4.33 ± 0.33 for non‐incubated PET and 4.43 ± 0.47 for the abiotic control (Figure 1B, Table 3). In contrast, sheets incubated with strain BI2 did not differ significantly from the controls (4.16 ± 0.34; 5.0%; *p* = 0.356). The ester index (A_1242_/A_1409_) followed the same trend, whereas no consistent change was detected in the crystallinity‐associated band at 1340 cm^−1^ across treatments.

**Table 3:** ATR‐FTIR semi‐quantitative analysis of PET sheets after 1 month of incubation.

Group	Description	*n*	Carbonyl index (mean ± SD)	Ester index (mean ± SD)	Δ Carbonyl index (%)	*p* versus control	Significance
P	PET, non‐incubated (initial control)	2	4.334 ± 0.326	4.511 ± 0.314	—	—	Control
C	PET abiotic control	2	4.429 ± 0.474	4.555 ± 0.271	—	—	Control
BI2	PET incubated with strain BI2	6	4.164 ± 0.343	4.388 ± 0.343	5.0	0.355	n.s.
16P	PET incubated with strain 16P	6	3.668 ± 0.153	3.820 ± 0.180	16.3	0.017	*
19A	PET incubated with strain 19A	6	3.727 ± 0.245	4.001 ± 0.421	14.9	0.019	*
BI8	PET incubated with strain BI8	6	3.609 ± 0.087	3.716 ± 0.107	17.6	0.017	*

*Note:* Δ Carbonyl index (%) = reduction relative to the mean of the pooled PET controls (P + C); n.s., not significant.

* *p* < 0.05.

It is worth noting that the two experimental measurements were not fully concordant for all strains: whereas 16P and BI8 showed both significant mass loss and a significant reduction of the carbonyl index, strain BI2 produced measurable mass loss without a detectable change in the ester carbonyl signal, and strain 19A showed the opposite pattern. Because the mass loss reflects the overall removal of material from the sheet, which may include physical erosion or loss of low‐molecular‐weight components, whereas the carbonyl index specifically tracks cleavage of the ester bond, this partial divergence is expected and indicates that the two approaches capture complementary aspects of the polymer's alteration.

### 3.3 Characterization of the Isolates From Genomic Data and Functional Annotation

The use of hybrid WGS allowed us to obtain complete and near‐complete genome sequences for all four strains, creating circular chromosomes with recognizable GC‐skew patterns, closely related to complete genomes (Figure S3). As for completeness, evaluated by CheckM2, all genomes showed high‐quality parameters (completeness > 90%, contamination < 5%, see Table 4). According to GTDB‐tk, the four genomes corresponded to a previously characterized species, identified with ANI screen only: *Neobacillus thermocopriae* (strain 19A), *Bacillus licheniformis* (strains 16P and BI2), and *Brevibacillus agri* (strain BI8). The longest contigs from *B. licheniformis* BI2 and 16P strains (isolated from *Baños del Inca* and *El Tragadero,* respectively), when compared (Figure S4) were shown to be almost identical, with the exception of a region present only in the BI2 strain, suggesting a very close resemblance between them and a potential clonal origin. Both *B. licheniformis* and *B. agri* Peruvian strains exhibited chromosomal‐level similarity with their type strain references (Figure S4). The *N. thermocopriae* isolate 19A could not be compared with its type strain since its assembly (Strain: SgZ‐7, accession GCA_010975035.1) was only available as contigs (*n* = 54, accession JAAIUV010000000).

**Table 4:** Main genomic statistics of the isolates from geothermal sources.

Strain	Genome length (bp)	Number of contigs	Longest contig length (bp)	GC%	Number of CDS	Checkm2 completeness (General)	Checkm2 contamination	Checkm2 completeness (Specific)
*Bacillus licheniformis* 16P	4,210,399	3	4,080,612	46.22	4295	100	0.03	99.99
*B. licheniformis* BI2	4,210,436	1	4,210,436	46.22	4291	100	0.03	99.99
*Neobacillus thermocopriae* 19A	3,467,457	2	3,466,052	37.99	3187	100	0.46	99.99
*Brevibacillus agri* BI8	5,655,312	1	5,655,312	53.94	5253	99.9	2.12	100

**Figure 2:**
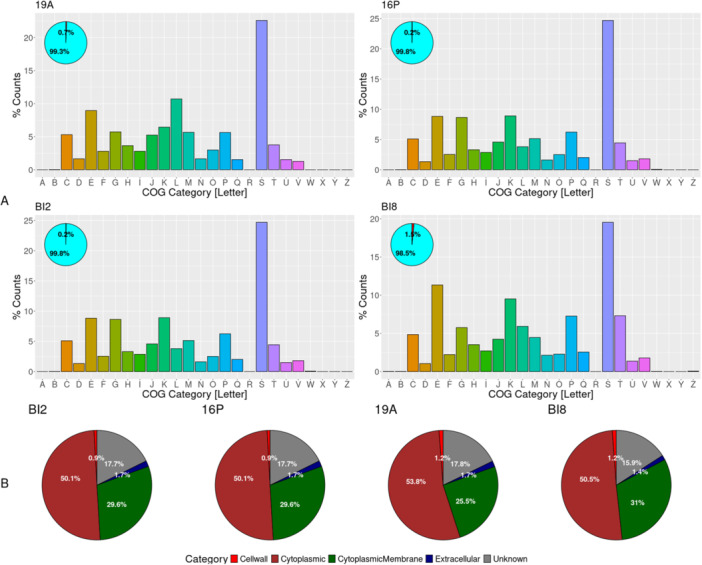
Annotation profiles from the predicted CDS from the sequenced Peruvian strains. (A) COG Functional profiles. Each strain was classified using the COG letter categorization criterion (available in https://ftp.ncbi.nih.gov/pub/COG/COG2020/data/fun-20.tab) and the ratio of classified (cyan) versus unclassified proteins (red) is represented in the pie charts in the upper left region of each barplot. (B) Subcellular localization profiles via PsortB 3.0. Each pie chart represents the predicted options for the Gram‐positive localizations for each predicted ORFeome.

The COG category profile of the four genomes showed a very low set of proteins without classification (range: 0.2%−1.5%, Figure 2A) and a very large set of proteins predicted into the S category (*Function unknown*, range: 19.54%−24.70%, Figure 2A). Among the proteins detected in the S category, EggNOG predicted activities associated with carboxylesterases (EC 3.1.1.1), triacylglycerol lipases (EC 3.1.1.3), and 2‐succinyl‐6‐hydroxy‐2,4‐cyclohexadiene‐1‐carboxylate synthases (EC 4.2.99.20); this latter enzyme, despite its indicated activity, exhibited similarity to a reported protein from a *Caldibacillus (Bacillus) thermoamylovorans* strain with esterase activity involved in the degradation of PET (Yan et al. 2024). This finding suggests that the S category contains numerous proteins with predictable enzymatic activities. On the other hand, all four genomes contained a gene potentially encoding a lysophospholipase (EC 3.1.1.5, COG category I). However, functional annotation did not find any genes for a laccase (EC 1.10.3.2) or a cutinase (EC 3.1.1.74) in any of the four strains.

Since several enzymes must act on the external side of the cell, the subcellular localization of the predicted proteins in the genomes was predicted (Figure 2B). As expected, a relatively reduced set of proteins were found to be predicted to be localized in the cell wall (range: 0.9%−1.2%, Figure 2B) or predicted to be released into the extracellular medium (range: 1.4%−1.7%, Figure 2B). Approximately 25%−29% of the proteins were predicted to be located in the cytoplasmic membrane (and, expectedly, most of them associated with transport processes) and 15%−17% of the proteins could not be predicted into a defined localization (category “Unknown”).

### 3.4 Phylogenetic Analysis of the 16S rRNA Genes of the Peruvian Strains

All 4 strains contained several copies encoding 16S rRNA genes, comprising 8 (strains 16 P and BI2), 11 (19 A), or 13 (BI8), indicating multiple copies. Inside each genome, all copies were virtually identical. Phylogenetic analysis in comparison with other 16S rRNA gene sequences from related bacteria (Figure S5) confirmed the placement of each strain inside different organisms and genera: copies from 16P and BI2 were at least 99.50% identical to the 16S rRNA gene of *B. licheniformis* strain ATCC 14580 (accession NR_074923, length: 1545 bp); gene copies from BI8 strain were at least 99.50% identical to the reference 16S rRNA gene from *B. agri* strain NBRC 15538 (accession NR_113767.1, length: 1462 bp). Finally, all gene copies from strain 19A were at least 99.4% identical to the rRNA gene from *N. thermocopriae* strain SgZ‐7 (NR_109664.1) despite close resemblance to the gene from other species such as *Neobacillus sedimentimangrovi* strain FJAT‐2464 16S (accession NR_181062.1). This latter behavior was also observed in the phylogenetic tree, in which all copies of isolates were detected in the same clade as their proposed relatives (Figure S5). Intraspecific relationships were supported in clades with high branch support by both aLRT or ultrafast bootstrap values: 100/100 for the 19A clade, 92/87 for the BI8 clade, and 96.9/98 for the BI2/16P clade.

### 3.5 Other Predicted Features From the Peruvian Isolates

In order to explore the potential antimicrobial resistance mechanisms in the sequenced Peruvian isolates, we compared the potential ORFeome against the CARD database (via the *RGI main* tool). From this analysis, we found that the four genomes contained different markers, with certain common features (Table S2). In all strains, multiple copies of genes encoding D,D‐carboxypeptidases (*vanY*‐like) were found. This enzyme is involved in the inhibition of glycopeptide antibiotics such as vancomycin or teicoplanin, via the modification of the natural binding target of the antimicrobial compounds: the terminal moieties of the Lipid II intermediates in the peptidoglycan layer maturation process (Stogios and Savchenko 2020). Additionally, the analysis also found candidates for antibiotic efflux pumps involved in small multidrug resistance (SMR) in the four isolates, as well as a fosfomycin thiol transferase in all isolates but 19A. In the *B. licheniformis* strains, a BcIII class A‐*Bacillus cereus* Bc beta‐lactamase marker was found, involved in beta‐lactam resistance via breakdown of a number of penicillins and cephalosporins (Torkar and Bedenić 2018).

In order to predict the potential for secondary metabolite biosynthesis in the Peruvian strains, we compared their complete annotation against the AntiSMASH database (Table S3). Using this tool, we found that these strains contained a set of metabolites involved in environmental adaptation and defense mechanisms. All those four strains were predicted to contain: (a) a terpene biosynthetic cluster (containing a sporulenol synthase, catalyzing the conversion from sporulenol to tetraprenyl‐ beta‐curcumene); (b) a type‐III polyketide synthase biosynthetic cluster, associated with the alpha‐pyrone synthesis polyketide synthase‐like activity, similar to Pks11, a protein involved in the biosynthesis of methyl‐branched alkylpyrones as in *Mycobacterium tuberculosis* (Gokulan et al. 2013); (c) a cluster probably involved in the biosynthesis of a terpene precursor, containing a putative farnesyl diphosphate synthase; and (d) a mixed cluster potentially involved in the biosynthesis of a non‐ribosomal peptide metallophore and/or a non‐ribosomal peptide synthetase. *B. licheniformis* strains contained another biosynthetic cluster for terpene precursors, containing a hypothetical protein related to a member of the polyprenyl synthetase family (COG0142, Geranylgeranyl pyrophosphate synthase). *B. licheniformis* strains contained also a potential cluster involved in the biosynthesis of a Beta‐lactone containing protease inhibitor, and for the synthesis of a Lasso peptide (ribosomally synthesized and post‐translationally modified peptides with antibacterial activity, Mucha et al. 2025; Maksimov et al. 2012). Strain BI8 contained distinctive features absent in the others, such as a potential cluster involved in the biosynthesis of a Opine‐like zincophore (containing two central genes encoding a putative staphylopine dehydrogenase and a putative methylase involved in staphylopine biosynthesis); BI8 strain also contained a distinctive cluster involved in the biosynthesis of a cyclic lactone autoinducer peptide, compound involved in intercellular communication via quorum sensing in some Gram‐positive bacteria such as *Staphylococcus aureus* (Wang et al. 2015).

Finally, another overall aspect to cover in the functional annotation is the potential repertoire of carbohydrate‐active enzymes (CAZymes). In order to obtain the CAZyme profile, the ORFeome of the Peruvian strains was analyzed by run_DBCAN against the CAZy repository from November 2025 (Table S4). The distribution patterns reveal distinct carbohydrate metabolic capabilities, possibly reflecting differential ecological adaptations. For example, as expected, the two *B. licheniformis* strains (16P and BI2) had identical profiles. These two strains showed a predicted cellulolytic machinery, reflected by the presence of variants of the glycoside hydrolase families 5 and 9, associated with cellulase (EC 3.2.1.4) and cellulose 1,4‐β‐cellobiosidase (EC 3.2.1.176) activities (GH5_4, CBM3 + GH5_2, CBM3 + GH9). These *B. licheniformis* strains also contained distinctively copies for a repertoire of GH43 family arabinofuranosidases: EC 3.2.1.55 (alpha‐L‐arabinofuranosidase) represented by GH43_10 and GH43_12; and EC 3.2.1.99 (arabinan endo‐1,5‐alpha‐L‐arabinanase) represented by GH43_5 and GH43_4. Notably, these activities may be involved in hydrolyzing α−1,3‐arabinofuranosyl residues from xylose units in plant cell wall hemicelluloses (Ahmed et al. 2013). Additionally, these strains contained GH51 family members, potentially representing another member representative of the EC 3.2.1.55 activity (Dos Santos et al. 2018). This analysis also found CE12/CE7 representatives in 16P and BI2 and those may be involved in beta‐xylosidase activities (EC 3.2.1.72), as well as the presence of multiple polysaccharide lyase (PL) families (PL1_5, PL1_6, PL1_8, PL3_1, PL9_2, PL11_1, and PL26) indicates specialized pectin degradation capabilities. In strains 16P, BI2, and 19A, genes encoding a potential alpha‐glucosidase (EC 3.2.1.20, GH13_31) were found. Strain BI8 contained a putative member of the GH15 family (CAZyme absent in the other three). Activities belonging to the GH15 family are commonly recognized as alpha‐glucosidase acting on amylose or trehalose. The 19A strain uniquely possesses GH2 (further predicted as a β‐galactosidase, EC 3.2.1.23), GH36 (α‐galactosidase, EC 3.2.1.22), and a copy of a GH13 subfamily member mostly proposed as an α‐glucosidase. Thus, the predicted repertoire of CAZymes suggests that these strains have different patterns of carbohydrate management and use.

### 3.6 Identification of PET‐Related Hydrolase Homologs by Sequence Similarity and Structure‐Based Analysis

As previously mentioned, experimental analysis of the four Peruvian strains suggested that those organisms harbored genes encoding potential esterases. However, the need for a more specific search, using highly curated reference databases, was performed to have a better approach to the search of PETases and related hydrolases. In order to make a first approach in searching for potential candidates, we compared the set of PAZy sequence entries associated with PET degradation against the predicted ORFeome of the four strains. This search found potential candidates related to previously studied proteins: in *B. licheniformis* strains, proteins showing noticeable similarity (*E*‐value < 10^−17^) were similar to UNZ81747.1, a protein annotated as a beta‐lactamase (COG1680) with hydrolase activity from a *Dehalococcoidia* enrichment culture obtained from geothermal areas of the volcanic island of Ischia (Gulf of Naples, Italy) (Distaso et al. 2023). Among these strains, a protein with significant similarity was found, similar to WP_034767800, an annotated esterase lipase (COG1647) found in *Caldibacillus thermoamylovorans* (Yan et al. 2024); both 16P and BI2 strains also contained similar proteins to ADH43200 (*E*‐value < 10^−67^; an intracellular p‐nitrobenzylesterase from the COG2272 family, previously detected and evaluated in a *Bacillus subtilis* strain, Ribitsch et al. 2011), WP_041846030 (*E*‐value < 10^−82^; another esterase from *C. thermoamylovorans*, Yan et al. 2024), APJ12152 (*E*‐value < 10^−70^, a lipase of the COG1075 family, obtained from *Bacillus safensis*, Vidal et al. 2024), CEE00769.1 (*E*‐value < 10^−180^, the annotated 2‐succinyl‐6‐hydroxy‐2,4‐cyclohexadiene‐1‐carboxylate synthase from *C. thermoamylovorans*, mentioned above), and WP_108898647.1 (*E*‐value < 10^−97^, another *C. thermoamylovorans* esterase, Yan et al. 2024). Interestingly, in the 19A strain, predicted proteins also exhibited notable similarity to UNZ81747, CEE00769, and WP_108898647, mentioned before. In BI8 strain, genes with noticeable similarity were found to WP_034767800, UNZ81747, CEE00769 (all of these three mentioned above) and RLI42440, a COG1647 family esterase previously found in a non‐cultivated *Candidatus* Bathyarchaeota archaeon, recovered by metagenomics (Perez‐Garcia et al. 2023). Considering these hits as potential candidates for PET hydrolases, the next step was performed on the basis of structural comparisons.

**Figure 3:**
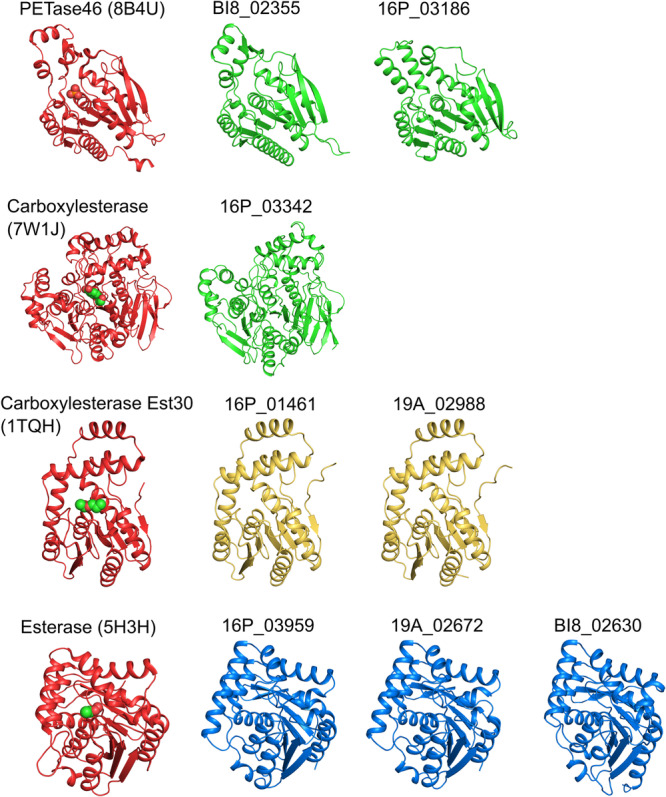
Structural comparison between reference PET‐related hydrolases and putative homologs identified in this study. Representations of experimentally characterized esterases and PET‐related hydrolases (left, red) and their corresponding putative homologs identified by structure‐based homology searches (right, colored). Reference structures include PETase46 (PDB: 8B4U), a carboxylesterase associated with (tere)phthalate ester hydrolysis (PDB: 7W1J), carboxylesterase Est30 (PDB: 1TQH), and the esterase EaEST (PDB: 5H3H). Putative homologs were modeled using AlphaFold 3. All structures show a conserved α/β‐hydrolase fold.

Structure‐based homology searches using *Foldseek* allowed the identification of several candidate enzymes exhibiting significant structural similarity to experimentally characterized esterases and PET‐related hydrolases (Figure 3). The selected candidates clustered into three main groups based on their closest structural homologs: (i) PETase46‐like enzymes (PDB: 8B4U), (ii) carboxylesterases associated with (tere)phthalate ester hydrolysis (PDB: 7W1J), and (iii) classical bacterial esterases and carboxylesterases (PDB: 1TQH and 5H3H). All identified candidates adopt the canonical α/β‐hydrolase fold, characterized by a central β‐sheet flanked by α‐helices, and display a catalytic architecture compatible with serine hydrolase activity (Ozhelvaci and Steczkiewicz 2025; Wei et al. 2022).

**Figure 4:**
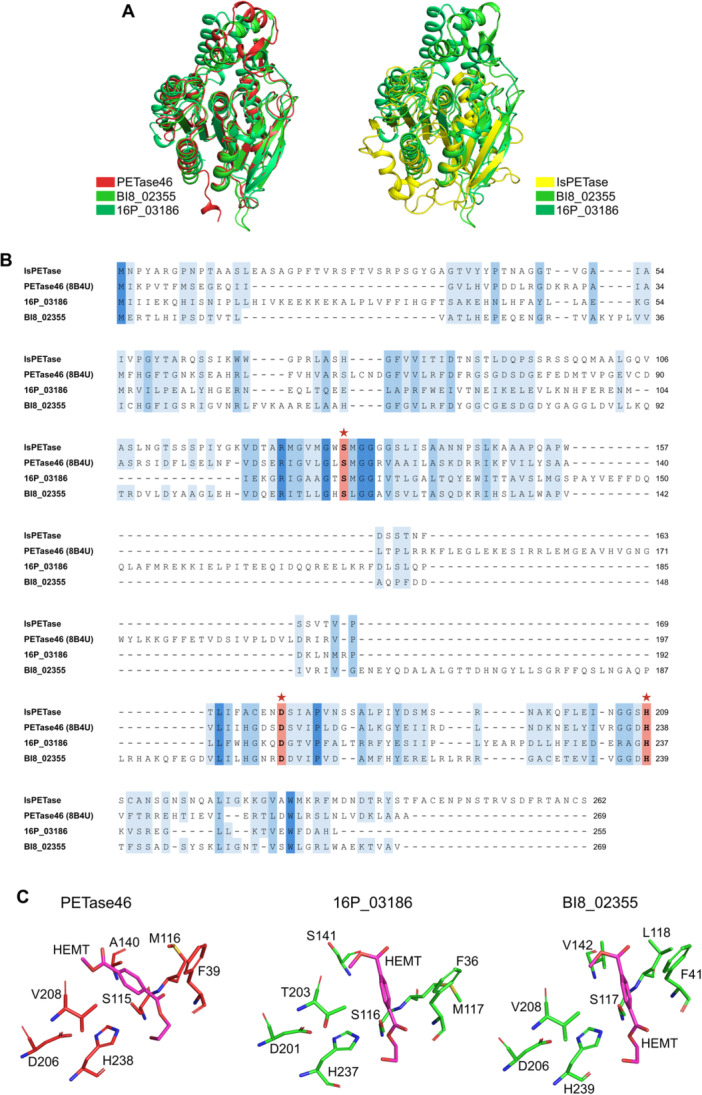
Structure‐based comparison of the PETase‐like candidates BI8_02355 and 16P_03186 against benchmark PET hydrolases. (A) Structural superposition of the candidates onto PETase46 (PDB: 8B4U) and onto IsPETase (PDB: 5XJH); BI8_02355 and 16P_03186 are shown in both panels. (B) Structure‐guided multiple sequence alignment of the two candidates with IsPETase and PETase46. Residues are shaded by conservation, and the catalytic triad is marked with red stars, corresponding to Ser160, Asp206, and His237 in IsPETase; the nucleophilic serine lies within the canonical G‐x‐S‐x‐G motif of the nucleophile elbow. (C) Active‐site view of the docked complexes of PETase46, 16P_03186, and BI8_02355 with HEMT, a PET‐derived ligand observed in the crystal structure of *I. sakaiensis* PETase (PDB: 5XH3). Residues of the catalytic triad and of the surrounding substrate‐binding environment are shown as sticks.

Two candidates, BI8_02355 and 16P_03186, were identified as close structural homologs of PETase46 (PDB: 8B4U), an enzyme experimentally demonstrated to hydrolyze PET‐derived substrates such as BHET and MHET (Perez‐Garcia et al. 2023). Structural superposition revealed a high degree of conservation in the overall fold and in the geometry of the catalytic active site (Figure 4). Because these two candidates (BI8_02355 and 16P_03186) represented the group most closely related to an experimentally validated PET hydrolase, this cluster was selected for a more detailed evaluation of catalytic‐site conservation. Therefore, *Is*PETase (the archetypal, experimentally characterized PET hydrolase from *I. sakaiensis*) was incorporated as a benchmark reference (Chen et al. 2022). Although *Is*PETase was not among the structural homologs retrieved by Foldseek, its well‐established structural relationship with PETase46 allowed its inclusion, and both candidates superimposed closely onto the *Is*PETase scaffold as well (Figure 4A).

A structure‐guided multiple sequence alignment of both candidates against the benchmark PET hydrolases PETase46 and *Is*PETase was subsequently performed to examine the conservation of catalytic residues. Both BI8_02355 and 16P_03186 retained the complete Ser‐His‐Asp catalytic triad, corresponding to Ser160, Asp206, and His237 in *Is*PETase, with the nucleophilic serine located within the canonical G‐x‐S‐x‐G motif of the nucleophile elbow (Figure 4B) (Chen et al. 2022; Han et al. 2017). Given the conservation of the catalytic triad, the capacity of the candidate active sites to accommodate a PET‐derived substrate was next assessed by molecular docking. Molecular docking analyses for PETase46 were first performed using HEMT, a PET‐derived ligand observed in the crystal structure of *I. sakaiensis* PETase (PDB: 5XH3). The same ligand and docking parameters were subsequently applied to BI8_02355 and 16P_03186. In the docked complexes, HEMT was positioned with its scissile carbonyl adjacent to the nucleophilic serine and stabilized by the surrounding hydrophobic and aromatic residues lining the binding cleft (Figure 4C), consistent with the substrate‐binding mode described for characterized PET hydrolases. Overall, the conservation of the catalytic triad together with a compatible substrate‐binding geometry supports the classification of BI8_02355 and 16P_03186 as putative PET‐hydrolase candidates, although these in silico observations do not by themselves constitute evidence of PET‐hydrolytic activity.

**Figure 5:**
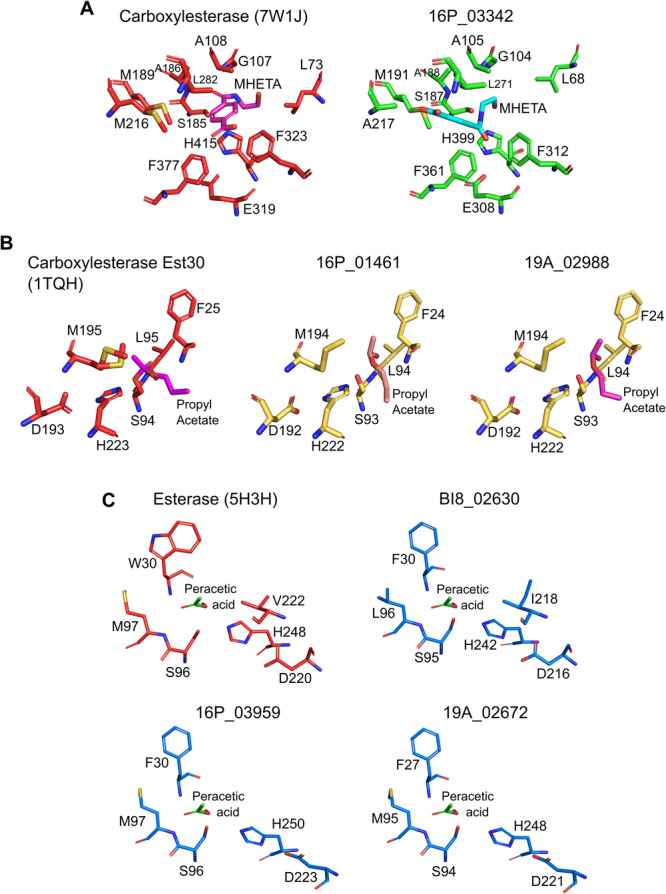
Active‐site comparison and ligand‐binding analysis of the auxiliary carboxylesterase and esterase homologs. (A) Comparison of the active‐site region of a carboxylesterase involved in (tere)phthalate ester hydrolysis (PDB: 7W1J) and its putative homolog 16P_03342. Docking was performed using MHETA, the ligand co‐crystallized with the reference structure. (B) Comparison of the active‐site regions of carboxylesterase Est30 (PDB: 1TQH) and its putative homologs 16P_01461 and 19A_02988, using the co‐crystallized ligand propyl acetate (magenta) to evaluate conservation of catalytic geometry and substrate accommodation. (C) Structural superposition of the esterase EaEST (PDB: 5H3H) and the putative homologs BI8_02630, 16P_03959, and 19A_02672. Owing to the small size of the co‐crystallized ligand (peracetic acid), no docking was performed for this group; instead, conservation of the catalytic environment was assessed from the spatial arrangement of the active‐site residues. In all panels, key catalytic and substrate‐binding residues are shown as sticks.

A second group of candidates, represented by 16P_03342, showed structural similarity to a carboxylesterase experimentally characterized for (tere)phthalate ester hydrolysis (PDB: 7W1J) (Figure 5A) (von Haugwitz et al. 2022). Docking simulations were performed using MHETA, the ligand co‐crystallized with the reference structure. Docking of MHETA into 16P_03342 resulted in ligand orientations comparable to those observed in the experimental structure, and structural comparison revealed conservation of the catalytic serine motif and residues lining the substrate‐binding pocket, including hydrophobic and aromatic residues implicated in substrate stabilization (Figure 5A). The conserved catalytic residues of this homolog are highlighted in the corresponding sequence alignment (Figure S6).

Additional candidates were identified as homologs of carboxylesterase Est30 (PDB: 1TQH) (Liu et al. 2004) and esterase *Ea*EST (PDB: 5H3H) (Lee et al. 2017) (Figure 5). For the Est30 homologs (16P_01461 and 19A_02988), docking analyses were performed using propyl acetate, the ligand co‐crystallized with the reference structure, allowing evaluation of the conservation of catalytic geometry and substrate accommodation (Figure 5B). For the *Ea*EST‐like homologs (BI8_02630, 16P_03959, and 19A_02672), molecular docking was not performed due to the small size of the co‐crystallized ligand (peracetic acid, PDB: 5H3H). Instead, structural superposition analyses were conducted, revealing conservation of the catalytic environment (Figure 5C). For all these auxiliary esterase candidates, the catalytic residues are highlighted in the corresponding sequence alignments (Figure S7); however, given that these enzymes belong to broad esterase/carboxylesterase families rather than validated PET hydrolases, their conserved catalytic architecture is reported here without implying direct activity on PET.

## 4 Discussion

The present study combines experimental screening, WGS, and structure‐based analyses to investigate the potential of thermophilic bacterial isolates from hot springs in Cajamarca‐Peru, to participate in PET biodegradation. Although PET mass loss under laboratory conditions was modest, all isolates were able to grow in minimal salt medium supplemented with PET as the sole carbon source, exhibited esterase activity in tributyrin assays, and three of them produced a significant reduction of the ester carbonyl signal at the PET sheet surface, as determined by ATR‐FTIR. Such limited degradation rates are consistent with previous studies showing that environmental isolates frequently exhibit slow PET hydrolysis in the absence of polymer pretreatment or optimized catalytic conditions (Charnock 2021; Wei and Zimmermann 2017; Hadad et al. 2005). These findings indicate that, while PET utilization under the tested conditions is not highly efficient, the isolates possess intrinsic metabolic features compatible with polyester interaction. ATR‐FTIR analysis provided chemical support for this interaction. After normalization to an internal aromatic reference, sheets incubated with strains 16P, 19A, and BI8 showed reduction of the ester carbonyl index relative to both the initial PET and the abiotic control, whereas strain BI2 did not differ from the controls. The inclusion of an abiotic control indicates that these changes cannot be attributed to thermal aging or abiotic hydrolysis of the polymer. The absence of detectable changes in the crystallinity‐associated band suggests that the modification was restricted to the sheet surface, which is consistent with the low mass loss values and with the semi‐crystalline, solid nature of the substrate. Some residual biological material was detected on the incubated sheets, which may also contribute to the O‐H/N‐H stretching region; therefore, these spectral changes are interpreted as evidence of preferential ester modification rather than as identification of specific hydrolysis products. Chromatographic quantification of soluble degradation products would be required for that purpose.

Morphological and physiological characterization revealed Gram‐positive, rod‐shaped, endospore‐forming bacteria belonging to the order Bacillales. Members of this group, particularly *Bacillus* species, are widely reported in plastic‐associated environments and are recognized for their metabolic versatility, stress tolerance, and ecological adaptability (Valdez‐Nuñez and Rivera‐Jacinto 2024; Vidal‐Verdú et al. 2022). The observed variability in colony morphology under identical culture conditions likely reflects adaptive plasticity rather than direct evidence of PET degradation. Environmental constraints such as nutrient limitation, osmotic stress, and elevated temperature are known to induce morphological and physiological shifts that enhance bacterial persistence and survival (van Teeseling et al. 2017; Kysela et al. 2016). In this context, geothermal environments may select for organisms with robust stress responses and versatile enzymatic systems capable of interacting with recalcitrant substrates. Growth at elevated temperatures is particularly relevant in the context of polyester degradation. PET hydrolysis is known to benefit from temperatures approaching the polymer's glass transition temperature (Tg), where increased chain mobility enhances enzymatic accessibility. Engineering efforts aimed at improving PET hydrolases have demonstrated that catalytic efficiency can increase significantly at elevated temperatures near Tg (Tournier et al. 2020; Austin et al. 2018). Although enzyme stability and activity were not directly measured in the present study, the identification of PET‐related hydrolases in bacteria adapted to growth at 50°C suggests potential for favorable thermal performance compared with mesophilic counterparts.

Genome mining against plastic‐oriented databases identified a heterogeneous repertoire of hydrolases, including esterases and carboxylesterases previously associated with aromatic ester and plastic degradation. Rather than revealing a single canonical PETase analogous to the enzyme described in *I. sakaiensis* (Perez‐Garcia et al. 2023; Yoshida et al. 2016), the genomic profiles suggest a distributed enzymatic system. This observation aligns with current models of microbial PET degradation, which emphasize multi‐step processes in which polymer depolymerization and intermediate processing are mediated by coordinated networks of hydrolases (Danso et al. 2019; Wei and Zimmermann 2017). Such distributed enzymatic repertoires may more accurately reflect natural biodegradation systems than single‐enzyme paradigms. It is important to note that, whereas strains BI2 and 16P were assigned to the same species, displayed nearly identical chromosomes and identical profiles of plastic‐active enzymes (PAZymes), the two strains differed in their interaction with PET: 16P exhibited markedly higher mass loss together with a significant reduction of the carbonyl index, whereas BI2 displayed measurable mass loss without a detectable change in the ester carbonyl signal. This divergence between two nearly clonal strains suggests that the genomic presence of predicted PAZymes is not sufficient to anticipate the extent or the chemical nature of PET modification, and highlights the need for improved methods to predict and experimentally validate such activities.

Structure‐based analyses further refined the functional interpretation of the genomic data. Foldseek searches and AlphaFold3 modeling identified candidate enzymes structurally related to experimentally characterized PET‐active hydrolases, including PETase46‐like proteins and terephthalate‐active carboxylesterases. The PETase‐like homologs BI8_02355 and 16P_03186 display conservation of the canonical α/β‐hydrolase fold and active‐site geometry relative to PETase46, including residues implicated in substrate binding and catalysis (Perez‐Garcia et al. 2023). Structural superposition and docking analyses using HEMT as a proxy PET‐related ligand support the conservation not only of the catalytic triad but also of the spatial arrangement of residues involved in substrate positioning and stabilization. These features mirror structural determinants described in both naturally occurring and engineered PETases, including active‐site accessibility and aromatic substrate accommodation (Tournier et al. 2020; Austin et al. 2018). The presence of such conserved structural elements in thermophilic isolates suggests evolutionary convergence toward polyester‐active architectures, even in organisms not previously characterized as dedicated PET degraders. In addition to PETase‐like homologs, the detection of a carboxylesterase structurally related to terephthalate‐active enzymes (von Haugwitz et al. 2022) indicates potential downstream processing capacity for PET‐derived intermediates such as MHET or related aromatic esters. Such enzymes have been proposed to act synergistically with PETases, preventing accumulation of partially hydrolyzed intermediates and improving overall degradation efficiency. Furthermore, multiple additional esterases exhibiting conserved catalytic environments were identified. Although these enzymes are unlikely to directly attack highly crystalline PET, their structural compatibility with ester hydrolysis suggests possible roles in processing low‐molecular‐weight intermediates generated during polymer breakdown. Together, these findings support a modular degradation framework in which thermophilic bacteria harbor coordinated enzymatic systems rather than relying on a single dominant PETase.

Importantly, the convergence between experimental observations (growth on PET‐containing media, esterase activity in tributyrin assays, and ester‐specific spectral changes detected by ATR‐FTIR), genomic identification of hydrolase‐encoding genes, and structural plausibility of substrate‐enzyme interactions provides a coherent multi‐level framework linking phenotype to molecular potential. Strains 16P and BI8 were consistently the most active across the three independent experimental measurements, and both encoded candidates structurally homologous to PETase46. While docking analyses and structural predictions do not constitute direct biochemical proof of PET hydrolytic activity, they establish a rational basis for targeted functional characterization. Future studies should focus on heterologous expression of the identified candidates, kinetic analyses against PET and PET‐derived substrates, and evaluation of temperature‐dependent catalytic efficiency to determine their true biotechnological potential. This study represents one of the first integrative genomic and structural explorations of PET‐related hydrolases in thermophilic bacteria isolated from Peruvian geothermal environments. By combining environmental microbiology, genome mining, and structural bioinformatics, this study expands the geographic and ecological landscape of candidate PET‐active enzymes and highlights the potential of understudied extreme habitats as reservoirs of thermally robust biocatalysts targeting synthetic polymers.

## Author Contributions

**Marco A. Rivera‐Jacinto:** conceptualization, data curation, formal analysis, funding acquisition, investigation, project administration, resources, supervision, validation, writing – original draft, writing – review and editing. **Claudia Rodríguez‐Ulloa:** conceptualization, data curation, formal analysis, investigation, project administration, methodology, supervision, validation, visualization. **Sara R. Briones‐Ramírez:** data curation, formal analysis, investigation, methodology, software, validation, visualization. **Betsy Quispe‐Cruz:** data curation, formal analysis, investigation, methodology, software, validation, visualization. **Sandra Carrasco‐Tarrillo:** data curation, formal analysis, investigation, methodology, software, validation, visualization. **Diego A. Leonardo:** conceptualization, data curation, formal analysis, investigation, methodology, software, validation, visualization, writing – original draft, writing – review and editing. **Juan P. Cardenas:** conceptualization, data curation, formal analysis, investigation, methodology, software, supervision, validation, visualization, writing – original draft, writing – review and editing.

## Ethics Statement

The authors have nothing to report.

## Conflicts of Interest

The authors declare no conflicts of interest.

## Supporting information


Supporting File 1

Supporting File 2

Supporting File 3

Supporting File 4

Supporting File 5

Supporting File 6

## Data Availability

The data that support the findings of this study are openly available in NCBI at https://www.ncbi.nlm.nih.gov/bioproject/?term=PRJNA1424230, reference number PRJNA1424230.
