# Supplementary material for: Experimental, Genomic, and Structural Evidence Supporting Putative PET‐Hydrolases in Thermophilic Bacteria Isolated From Hot Springs in Cajamarca, Peru
Source: Microbiologyopen. 2026 Aug 13;15(4):e70376. doi: 10.1002/mbo3.70376 (PMC13473705; doi:10.1002/mbo3.70376)
Supplement: Supplementary file 1 [file MBO3-15-e70376-s001.docx]

**Supplementary Table 1.** Phenotypic characteristics of bacterial strains during PET degradation assays.

| **Biodegradation assay** | **Strain** | | | |
| --- | --- | --- | --- | --- |
|  | **BI2** | **BI8** | **19A** | **16P** |
| Growth on solid medium + 0.0075% PET (colony diameter: mm) - 72 h | 6.5 | 1 | 1 | 5 |
| Growth on solid medium + 0.1% PET (colony diameter: mm) - 10 days | 22 | 4.5 | 3 | 7.5 |
| Growth in liquid medium + PET [0.77± 0.03 % (w/v)] | Positive | Positive | Positive | Positive |
| Percentage (%) of mass loss ± SD | 0.287 ± 0.032 | 0.449 ± 0.07 | 0.209 ± 0,008 | 0.598 ± 0.051 |
