# Supplementary material for: Experimental, Genomic, and Structural Evidence Supporting Putative PET‐Hydrolases in Thermophilic Bacteria Isolated From Hot Springs in Cajamarca, Peru
Source: Microbiologyopen. 2026 Aug 13;15(4):e70376. doi: 10.1002/mbo3.70376 (PMC13473705; doi:10.1002/mbo3.70376)
Supplement: Supplementary file 2 [file MBO3-15-e70376-s002.docx]

## **Supplementary data**

**Supplementary Figure 1. A. Tributyrin hydrolysis observed for each isolate.** Colonies at 72 h of growth: A1. strain BI2, A2. strain BI8, A3. strain 19A, A4. strain 16P. **B. Growth of isolates on PET agar.** Colonies at 72 h of growth: B1. strain BI2, B2. strain BI8, B3. strain 19A, B4. strain 16P.

**Supplementary Figure 2.** Percentage mass loss (%WL) of PET sheets following incubation with different bacterial strains, compared to the abiotic control (no bacterial inoculum). Data represent the mean ± SD (n = 3). Groups with different letters differ significantly (one-way ANOVA, F = 85.5, p < 0.001; Tukey's HSD test, α = 0.05).

**Supplementary Figure 3.** Circular genome map for the potentially complete chromosomes from the four strains obtained from the Peruvian geothermal sites. Each chromosome corresponded to the longest contig of each assembly. The notation of the color codes for the map are shown in the middle.

**Supplementary Figure 4.** Chromosome comparison between isolated genomes and reference strains. A. Genome alignment for the *Bacillus licheniformis* strains BI2 and 16P against the type strain of *B. licheniformis* (strain ATCC 14580, assembly GCF_034478925); B. Genome alignment for the *Brevibacillus agri* strain BI8 against the corresponding type strain (strain DSM 6348 , assembly GCF_004117055). The “_rc” indicates that the sequence was the reverse complementary version of the assembly.

*Continued on the next page.

*Continued on the next page.

*Continued on the next page.

*Continued on the next page.

**Supplementary Figure 5**. 16S Ribosomal RNA subunit-based phylogenetic tree using selected representatives from different groups related to the four Peruvian strains analyzed in his study. The tree was computed from the best hits from a BLASTN against the *refseq_rna* database in January 2026. Branch support values (obtained from the aLRT analysis) were put as nodes by color codes. The multiple copies found in each genome were included.

**Supplementary Figure 6. Multiple sequence alignment of PET-related hydrolase homologs and conservation of catalytic residues.** (A) Sequence alignment of the experimentally characterized carboxylesterase involved in (tere)phthalate ester hydrolysis (PDB: 7W1J) and its putative homolog 16P_03342. Conserved residues within the catalytic motif and surrounding regions are indicated, supporting structural and functional relatedness. Catalytically relevant residues are marked with asterisks, and conserved positions are highlighted according to residue identity.

**Supplementary Figure 7.** Sequence conservation in esterase and carboxylesterase homologs associated with PET-related intermediate processing. (A) Multiple sequence alignment of the experimentally characterized carboxylesterase Est30 (PDB: 1TQH) and its putative homologs 16P_01461 and 19A_02988. Conserved residues corresponding to the catalytic motif and substrate-interacting regions are highlighted, indicating preservation of key functional elements involved in ester bond hydrolysis. (B) Multiple sequence alignment of the esterase EaEST (PDB: 5H3H) and the putative homologs 16P_03959, 19A_02672, and BI8_02630. Conserved residues within the catalytic triad and surrounding active-site environment are indicated. Catalytically relevant residues are marked with asterisks, and conserved positions are highlighted according to residue identity.

**Supplementary Table 1.** Phenotypic characteristics of bacterial strains during PET degradation assays.

**Supplementary Table 2.** CARD markers found in the four strains obtained from the Peruvian geothermal sites. The ORFomes were analyzed by the *RGI main* tool from CARD with Strict hits.

**Supplementary Table 3.** Putative secondary metabolite biosynthetic clusters found in the four strains obtained from the Peruvian geothermal sites. The annotated genomes were analyzed by the *AntiSMASH* tool in “relaxed” mode.

**Supplementary Table 4.** Putative CAZymes found in the four strains obtained from the Peruvian geothermal sites. The annotated genomes were analyzed by the *runDBCAN* tool 4.1.4; genes predicted only by three simultaneous tools (HMMER, dbCAN_sub, DIAMOND) were considered.
